# Spatial clusters, social determinants of health and risk of COVID-19 mortality in Brazilian children and adolescents: A nationwide population-based ecological study

**DOI:** 10.1016/j.lana.2022.100311

**Published:** 2022-06-29

**Authors:** Victor Santana Santos, Thayane Santos Siqueira, Ana I. Cubas Atienzar, Maria Augusta Ricardo da Rocha Santos, Sarah Cristina Fontes Vieira, Aline de Siqueira Alves Lopes, José Rodrigo Santos Silva, Paulo Ricardo Martins-Filho, Luis Eduardo Cuevas, Ricardo Queiroz Gurgel

**Affiliations:** a Department of Medicine, Federal University of Sergipe, Lagarto, Brazil; b Health Sciences Graduate Program, Federal University of Sergipe, Aracaju, Brazil; c Department of Clinical Sciences, Liverpool School of Tropical Medicine, Liverpool, United Kingdom; d Department of Nursing, Federal University of Alagoas, Arapiraca, Brazil; e Division of Paediatric, Department of Medicine, Federal University of Sergipe, Aracaju, Brazil; f Department of Statistics and Actuarial Sciences, Federal University of Sergipe, Aracaju, Brazil; g Investigative Pathology Laboratory, Federal University of Sergipe, Aracaju, Brazil

**Keywords:** COVID-19, Spatial distribution, Social determinants of health, Mortality, Children, Adolescents, Brazil

## Abstract

**Background:**

Data regarding the geographical distribution of cases and risk factors for COVID-19 death in children and adolescents are scarce. We describe the spatial distribution of COVID-19 cases and deaths in paediatric population and their association with social determinants of health in Brazil.

**Methods:**

This is a population-based ecological study with a spatial analysis of all cases and deaths due to COVID-19 in Brazil among children and adolescents aged 0–19 years from March 2020 to October 2021. The units of analysis were the 5570 municipalities. Data on COVID-19 cases and deaths, social vulnerability, health inequities, and health system capacity were obtained from publicly available databases. Municipalities were stratified from low to very high COVID-19 incidence and mortality using K-means clustering procedures, and spatial clusters and relative risks were estimated using spatial statistics with Poisson probability models. The relationship between COVID-19 estimates and social determinants of health was explored by using multivariate Beta regression techniques.

**Findings:**

A total of 33,991 COVID-19 cases and 2424 deaths among children and adolescents aged 0–19 years were recorded from March 2020 to October 2021. There was a spatial dependence for the crude mortality coefficient per 100,000 population in the paediatric population aged 0–19 years (I Moran 0·10; *P* < 0·001). Forty municipalities had higher mortality rates, of which 20 were in states from the Northeast region. Seven spatial clusters were identified for COVID-19 mortality, with four clusters in the Northeast region and three in the North region. Municipalities with higher social inequality and vulnerability had higher COVID-19 mortality in the paediatric population.

**Interpretation:**

The main clusters of risk for mortality among children and adolescents were identified in municipalities in the North and Northeast regions, which are the regions with the worst socioeconomic indicators and greatest health disparities in the country. Our findings confirmed the higher burden of COVID-19 for Brazilian paediatric population in municipalities with higher social inequality and vulnerability and worse socioeconomic indicators. To reduce the burden of COVID-19 on children, mass immunisation is necessary.

**Funding:**

None.

**Research in context**

**Evidence before this study**

Although children and adolescents are the minority of COVID-19 cases, deaths occur in the paediatric population. Previous study showed an increased risk of mortality from COVID-19 in children younger than four years of age independent of comorbidities. In Brazil, COVID-19´s children mortality seems to be higher than in other countries. However, there is little information on how deaths in children and adolescents due to COVID-19 are related to social and economic characteristics. We searched PubMed, Scopus, Web of Science, Embase, medRxiv and bioRxiv on 19 February 2022 for published studies, without language restriction, that described the spatial distribution of COVID-19 deaths in paediatric population and their association to social vulnerability. We used the search terms “COVID-19”, “child”, “adolescent”, “mortality”, “spatial distribution” and “social vulnerability” and related synonyms. Only one study conducted in Mexico on the impact of environmental and individual factors on COVID-19 mortality was identified, but none on COVID-19-related deaths in children and adolescents and social determinants of health (SDH).

**Added value of this study**

We describe the spatial distribution of all COVID-19 cases and deaths in the paediatric population and their association with socioeconomic characteristics in Brazil from March 2020 to October 2021. The data provides evidence of the effects of geographic and regional inequities, health disparities and poverty on unfavourable outcomes of children and adolescents affected by COVID-19 in Brazil.

**Implications of all the available evidence**

COVID-19 cases and deaths were heterogeneously distributed across the Brazilian regions, with clusters mostly located in areas with a high degree of social vulnerability. Identifying the geographical areas at highest risk of exposure to adverse outcomes could be used to target interventions for mass testing, isolation of cases to mitigate the spread of the disease, mass immunization, as well as allocating the necessary health resources to prevent child deaths. Furthermore, our data corroborate children and adolescents constitute a priority group to receive COVID-19 vaccines.

Alt-text: Unlabelled box

## Introduction

Coronavirus disease-19 (COVID-19) was first detected in December 2019 and declared a global pandemic in March 2020,^1^ causing more than 5.9 million deaths by February 2022.^2^ The disease predominantly affects adults, and older individuals are more likely to have severe clinical presentations and risk of death.^3^ Although children and adolescents account for a minority of cases, and have more favourable disease outcomes, some develop severe acute clinical conditions^4^, ^5^, ^6^, ^7^ with hospitalization rates varying from 2·5% to 4·1%^7^, ^8^, ^9^ and case fatality rates (CFR) up to 8% of hospitalized children and adolescents.^10^ However, the epidemiology and risk factors for disease severity in children are not fully elucidated and whether incidence and outcomes are associated with social determinants of health (SDH),^6^^,^^11^ particularly from low- and middle-income countries.

Brazil was severely affected by COVID-19, with more than 28 million cases and 649,000 deaths reported by February, 2022.^2^ Although children represent only 1·9% of incident cases and 0·5% of deaths,^10^ these correspond to 23·6 deaths per million children, which is one of the highest mortality rates in the world.^12^ Previous reports indicated that children in the North and Northeast regions (the least developed regions of the country) or belonging to indigenous populations have a higher risk of death, independently of age and the presence of comorbidities.^7^^,^^13^ As the country has marked social inequalities, millions of people and children live in precarious living conditions.^14^ However, data regarding COVID-19 in children and adolescents are scarce, with a paucity on the risk factors for poor outcomes and their association with social determinants. We therefore report here an analysis of the association of the spatial distribution of COVID-19 cases and deaths among children and adolescents <20 years old in Brazil, and their relationship to SDH. The identification of children and adolescents at greatest risk of adverse outcomes could inform the development of targeted interventions to decrease their morbidity and mortality.

## Methods

### Study design

We conducted a population-based ecological analysis of the spatial distribution of all cases and deaths due to COVID-19 among Brazilian children and adolescents aged 0-19 years registered in the Influenza Epidemiological Surveillance Information System (SIVEP-Gripe) dataset from March 2020 to October 2021. All cases were confirmed by reverse transcription polymerase chain reaction (RT-PCR) assays for severe acute respiratory syndrome coronavirus 2 (SARS-CoV-2). The geographic units of analysis were the municipalities, and we included all the municipalities of the country. We examined the relationship between the municipalities' COVID-19 incidence, mortality and CFR, socioeconomic indicators and availability of health care resources. All analyses were performed considering the children and adolescents' residence data.

### Study area

Brazil has a geographic area of 8·5 million square kilometres, and ∼210 million population, of whom approximately 60 million are less than 20 years old.^15^ Brazil comprises 26 states and one federal administrative district and is subdivided into 5,570 municipalities. Illiteracy rates in people ≥15 years old is 6·6%, the Human Development Index (HDI) is 0·765 and infant mortality is 12·4 deaths per 1,000 live births.^16^

### Data sources and measures

Data were obtained from a variety of publicly available databases. The number of cases and deaths by COVID-19 was obtained from the SIVEP-Gripe dataset.^17^^,^^18^ SIVEP-Gripe is a deidentified nationwide public domain database established by the Brazilian Ministry of Health for the surveillance of acute respiratory distress syndromes. COVID-19 notifications became compulsory in March 2020, and the SIVEP-Gripe receives notifications of all cases from both public and private hospitals. Population data, stratified by age, were obtained from the Brazilian Institute of Geography and Statistics (IBGE; https://www.ibge.gov.br).

Detailed information on the filters used in SIVEP-Gripe is available in the Appendix (p. 1). Data for each municipality were used to calculate COVID-19 incidence and mortality coefficients per 100,000 population. CFR were estimated by dividing the number of deaths by the number of paediatric cases registered by the municipality.

Demographic and socio-economic data by municipality were obtained from the 2010 Brazilian Census.^19^ These included unemployment rates (%), illiteracy percentage, percentage of households with inadequate water supply, sewage or rubbish collection services and percentage of households with a per capita income below half the minimum salary, Municipal Human Development Index (MHDI) and the Gini index. The MHDI is composed of indicators from three dimensions of human development: longevity, education, and income. The MHDI and its domains (MHDI longevity, MHDI education, and MHDI income) range from 0 to 1, with values closer to 1 indicating higher human development. The Gini index measures the degree of income concentration in a population group and ranges from 0 to 1, with values closer to 1 representing higher income concentration.

The Social Vulnerability Index (SVI) was obtained from the Institute of Applied Economic Research.^20^ This index estimates the degree of vulnerability and social exclusion of a population and is composed of 16 social indicators comprising domains of urban infrastructure, human capital, and income and work. The SVI scores range from 0 to 1, and higher values indicate higher social vulnerability.^18^^,^^21^

We used the Ministry of Health's National Registry of Health Establishments (CNES) to assess the capacity of municipal-level health services to care for children and adolescents. We computed the number of pre-existing and new intensive care unit (ICU) beds, the number of paediatric hospital beds and of outpatient clinics. The ICU and hospital beds and outpatients’ clinics rates per 100,000 population were estimated using IBGE's 2020 population estimates by municipality. Data on the percentage of families covered by the Brazilian Family Health strategy, as well as physician and nurse coverage by municipality were obtained from CNES from March 2020 to October 2021. Detailed information on the demographic, socioeconomic, and healthcare indicators are described in Supplementary Table 1 (Appendix, p. 2).

### Data analysis

#### Spatial analysis

The spatial distribution maps were analysed in R software (version 4·1·2), using the cartographic base of Brazil available on the IBGE website.^22^ We mapped the incidence and mortality coefficients per 100,000 population and CFR stratified by age and by municipality. We applied the K-means clustering procedures following the Hartigan–Wong algorithm to stratify municipalities from low to very high COVID-19 incidence, mortality and CFR.^23^

Crude data rates were smoothed using Bayesian empirical local modelling to reduce the random variation of small areas and those with low frequencies. The Moran Global statistic was used to identify spatial autocorrelations, and when these were identified, we used the Local Index of Spatial Association (LISA). Scattering diagrams were generated to position the municipalities into quadrants (Q) and calculated the neighbouring municipalities average into Q1 (high/high: positive values and positive averages), Q2 (low/low: negative values and negative averages); Q3 (high/low: positive values and negative averages); Q4 (low/high: negative values and positive averages). The LISA Map departs from the Local Moran Index for the identification of different patterns of statistical significance (non-significant, 5% significance, 1% significance and 0·1% significance). The Moran Map only considers areas whose Moran indexes were significant (*P*-value <0·05).

We used the flexible spatial scan statistic with Poisson probability model with log likelihood ratio and 10 census areas as the maximum spatial cluster size to identify spatial clusters of the disease and estimate relative risks.^24^^,^^25^

#### Regression modeling

We examined the relationship between incidence, mortality and CFR and the social determinants of health. Initially the correlation between variables was examined using the Spearman rho test. As there was multicollinearity among several socioeconomic indicators, we used Principal Component Analyses (PCA) with the Varimax rotation and Kaiser normalization methods to reduce the dimension of the independent variables. The dependent variables (incidence, mortality and CFR) were modelled by Beta regression, assuming as independent variables the factors extracted from the PCA. A total of 21 independent variables were transformed into orthogonal factors. Factors with eigenvalue ≥1 were designated to compose the PCA. We observed the 5^th^ factor presented an eigenvalue equal to 1·0 and an accumulated variance of 71·6% (Supplementary Table 2; Appendix, p. 4). The components were distributed as follows:

PCA 1 - municipalities with a lower percentage of people living on low incomes and a lower percentage of unemployed people, lower SVI scores, lower SVI scores for human capital and work and income, a lower percentage of illiterate people, higher MHDI values for general, longevity, education and income.
PCA 2 - municipalities with lower pre-existing ICU rates, lower rates of new ICU beds in response to COVID-19, and lower average physicians’ and nurses’ coverage (lower than the average).
PCA 3 - municipalities with the lowest rate of outpatient clinics per 100,000 inhabitants and lowest percentage of family health teams.
PCA 4 - municipalities with higher Gini index (i.e., higher social inequality), higher SVI scores in infrastructure, higher percentage of households with inadequate piped water and sewage collection, and higher percentage of households with inadequate rubbish collection.
PCA 5 - municipalities with the lowest hospital bed rates.

#### Ethical considerations

Institutional review board approval and informed consent were not required because all data were obtained from public domain databases and were deidentified.

#### Role of the funding source

There was no funding source for this study. The corresponding author has full access to all the data in the study and had final responsibility for the decision to submit for publication.

## Results

A total of 33,991 COVID-19 cases and 2424 deaths were recorded among children and adolescents aged 0-19 years from March 2020 to October 2021. The spatial COVID-19 incidence distribution per 100,000 children <20 years old population had wide variation across municipalities, as shown in Supplementary Figure 1 (Appendix, p. 5). Three thousand and seven municipalities had incidence rates below 26 per 100,000 children <20 years old population; 196 municipalities had rates from 159 to 338; five between 947 and 3679 and only one municipality had rates higher than 3679 per 100,000 population. There was spatial dependence on the crude incidence rates (I Moran 0·12; *P* < 0·001) and 86 municipalities were classified as high risk for COVID-19 (Q1 Moran Map). The municipalities with the highest incidence rates were located in Sergipe (*n* = 22); Rio Grande do Sul (n = 15); Mato Grosso (*n* = 14) and Amazonas (*n* = 12) states.

**Figure 1:**
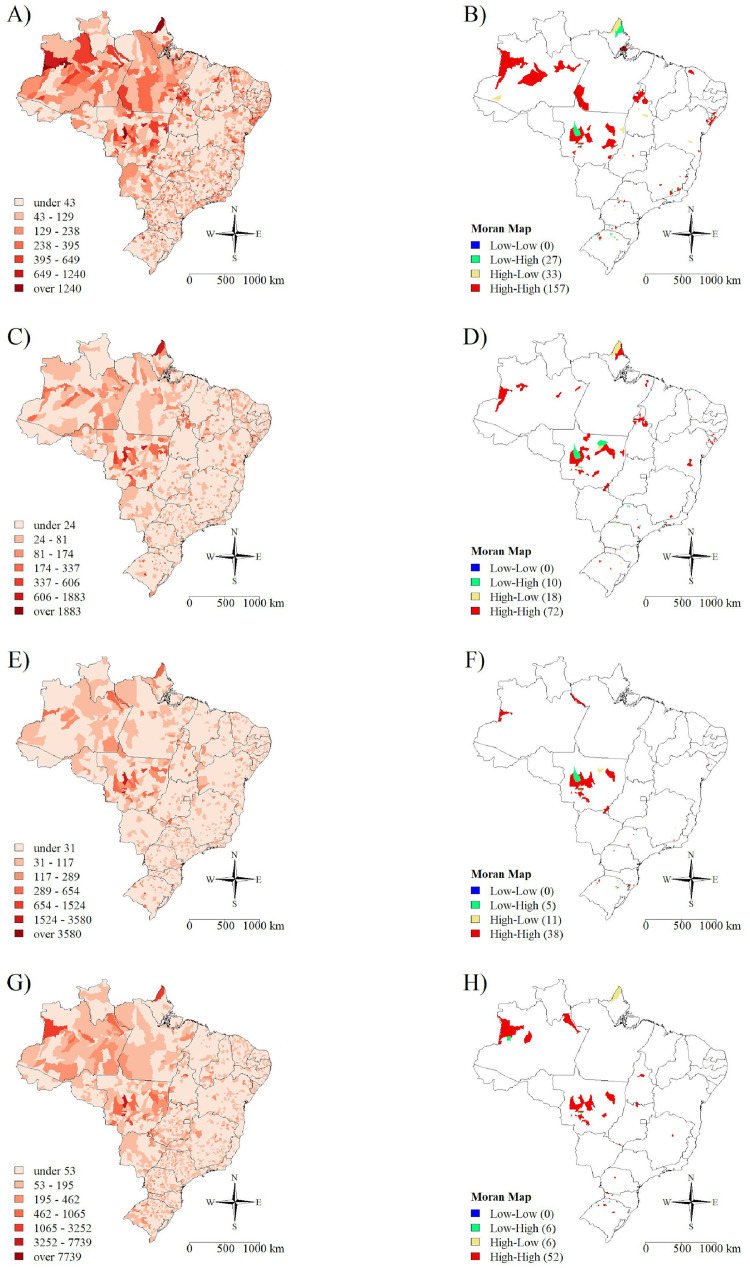
Spatial distribution of COVID 19 by age group. A) incidence rates per 100,000 population in children aged 0–4 years; B) Moran Map for children aged 0–4 years; C) Incidence rates per 100,000 population in children aged 5–9 years; D) Moran Map for children aged 5–9 years; E) incidence rates per 100,000 population for adolescents aged 10–14 years; F) Moran Map for adolescents aged 10–14 years; G) incidence rates per 100,000 population for adolescents aged 15–19 years; H) Moran Map for adolescents aged 15–19 years.

The spatial distribution of the COVID-19 incidence rate per 100,000 population by age group is shown in Figure 1 and Supplementary Table 3 (Appendix, p. 6). There was spatial dependence on the COVID-19 incidence rate for all age groups, with 157 municipalities having high incidence rates among children aged 0–4 years, of which 63 were in states from Northeast region, 33 were in the North region, 22 in the South region, 20 in the Southeast region, and 19 in the Central-West region. Seventy-two municipalities had high incidence rates among the 5–9 years age group, of which 27 were in the Northeast, 16 were in the North, and 14 were in the Central-West region. For the 10–14 years age group, 38 municipalities had high incidence rates, of which 14 were in the Central-West and 12 in the South regions. Lastly, 52 municipalities had high incidence rates among adolescents aged 15-19 years, with 24 municipalities located in the Southern region, 15 in the Central-West, and 11 in the North region.

**Figure 2:**
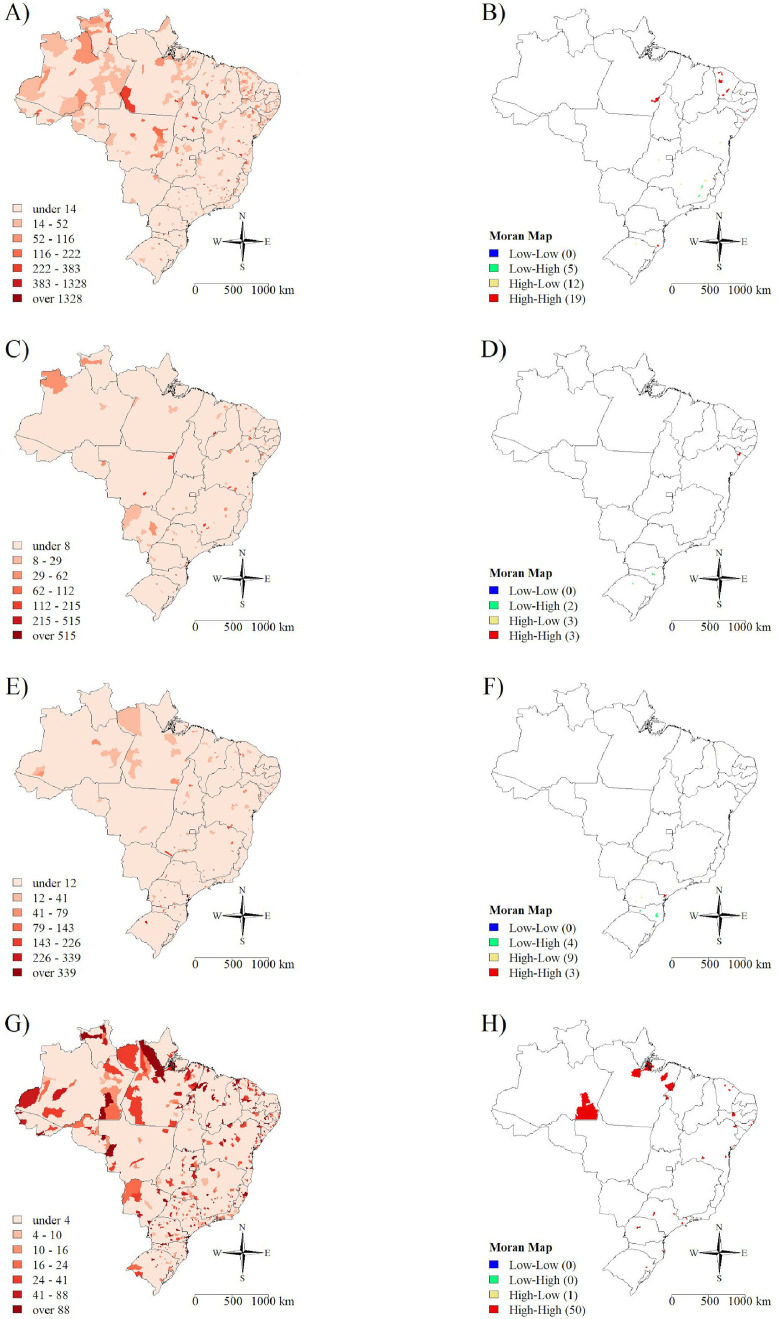
Spatial distribution of the COVID 19 mortality rate per 100,000 population according to paediatric age group in Brazil from March 2020 to October 2021. A) Children aged 0–4 years; B) Moran Map for Children aged 0–4 years; C) Children aged 5–9 years; D) Moran Map for Children aged 5–9 years; E) Adolescents aged 10–14 years; F) Moran Map for Adolescents aged 10–14 years; G) Adolescents aged 15–19 years; H) Moran Map for Adolescents aged 15–19 years.

There was also spatial dependence for the crude mortality coefficient per 100,000 population, as shown in Supplementary Figure 2 (Appendix, p. 9) (I Moran 0·10; *P* < 0·001). The stratum Q1 (high/high, corresponding to municipalities with high mortality rates which are surrounded by municipalities with high mortality rates) was composed of 40 municipalities, of which 20 were in the Northeast, eight in the South, and six in the Central-West and Southeast regions. Figure 2 and Supplementary Table 4 (appendix, p. 10) describes the spatial distribution of COVID-19 mortality rates by age group. Nineteen municipalities had high COVID-19 mortality rate among children aged 0–4 years old, of which 13 were in the Northeast and two in each of the North, Southeast and South regions. For children 5–9 years old, only three municipalities had high mortality and all three were in the Northeast region (all in Sergipe state). For children in the 10–14 age group, we identified three municipalities with high mortality rates; two in São Paulo and one in Santa Catarina states; while for the 15–19 age group we identified seven municipalities in Paraná (*n*= 3); São Paulo (*n*= 2); Rio Grande do Sul (*n*= 1) and Ceará (*n*= 1) states.

**Figure 3:**
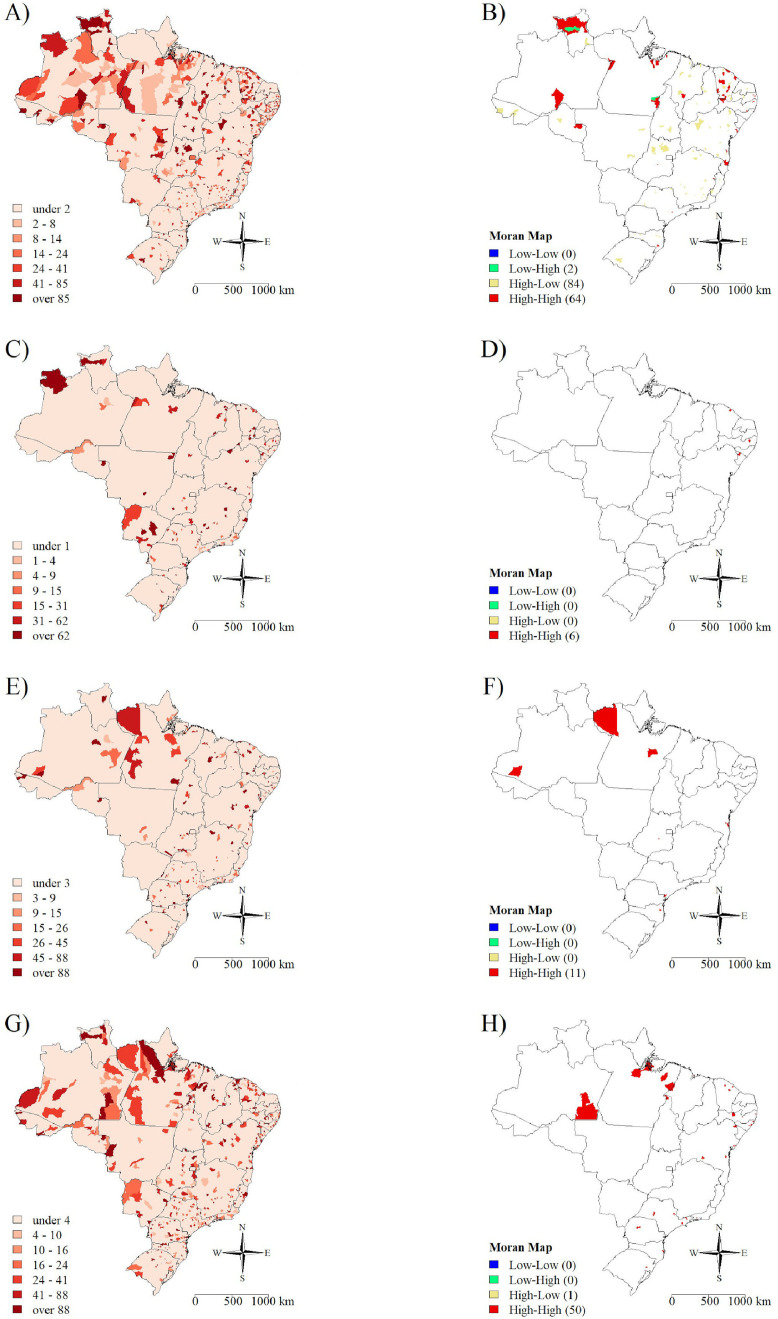
Spatial distribution of the COVID 19 case fatality rate per 100,000 population according to paediatric age group in Brazil from March 2020 to October 2021. A) Children aged 0–4 years; B) Moran Map for Children aged 0–4 years; C) Children aged 5–9 years; D) Moran Map for Children aged 5–9 years; E) Adolescents aged 10–14 years; F) Moran Map for Adolescents aged 10–14 years; G) Adolescents aged 15–19 years; H) Moran Map for Adolescents aged 15–19 years.

The spatial distribution of the crude COVID-19 CFR is shown in Supplementary Figure 3 (Appendix, p. 13). There was spatial dependence of the CFR with 94 municipalities having a high rate. Of these, 60 were in the Northeast, 15 in the North, 12 in the South, five in the Central-West, and two in the Southeast regions. Sixty-four municipalities had high CFR among children 0–4 years-old, of which 40 were in the Northeast and 17 in the North regions. Only six municipalities had high CFR in 5–9 years-old children, all in the Northeast region. Eleven municipalities had high CFR among adolescents aged 10–14 years, of which five were in the North region; and 50 had high CFR among adolescents aged 15–19 years, with 23 in the Northeast, 10 in the North, eight in the Southeast, and nine in the South region (Figure 3 and Supplementary Table 5 in the appendix p. 14).

**Figure 4:**
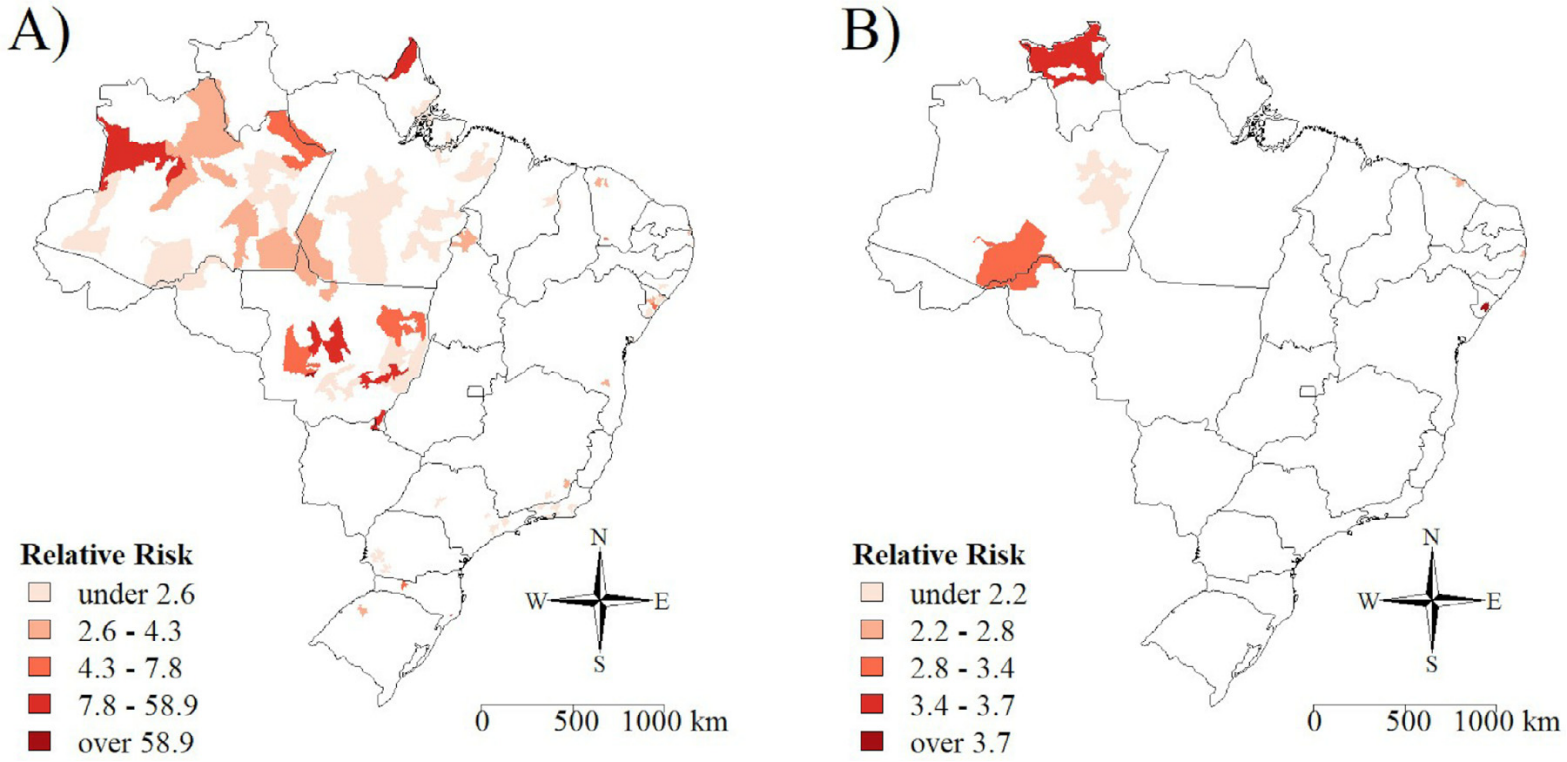
Spatial clusters of A) COVID-19 cases and B) COVID-19 deaths per 100,000 paediatric population (0–19 years) in Brazil from March 2020 to October 2021.

The spatial scan statistics identified spatial clusters with the highest COVID-19 incidence and mortality, as listed in Figure 4 and Supplementary Tables 6 and 7 (appendix p. 17 and 18, respectively). Fifty-six clusters were detected for COVID-19 incidence, which are distributed across all five regions, with 17 clusters in the North, 14 in the Northeast, 10 in the Southeast, 10 in the Central-West, and five in the South. Seven spatial clusters were identified for COVID-19 mortality, with four clusters in the Northeast and three in the North regions. The clusters with the highest risk of death were located in Sergipe (RR = 3.9), Bahia (RR = 3.9) and Roraima (RR = 3.5) states.

The correlation analysis shows the incidence, mortality and CFR for all paediatric populations (0–19 years) and age groups (Table 1) are associated with income inequity (Gini index), the social vulnerability index (overall and with the domains of urban infrastructure, human capital and work and income) and unemployment. There was a negative correlation between COVID-19 incidence, mortality and CFR rates and the percentage of the population living in households with inadequate water supply, sewage and waste collection services.

**Table 1:** Correlation between Social Determinants of Health and incidence of COVID-19, mortality and case fatality rate in Brazil according to paediatric age group in Brazil from March 2020 to October 2021.

Variable	0–4 years			5–9 years			10–14 years			15–19 years			0–19 years		
	Correlation coefficient			Correlation coefficient			Correlation coefficient			Correlation coefficient			Correlation coefficient		
	Incidence	Mortality	Case fatality rate	Incidence	Mortality	Case fatality rate	Incidence	Mortality	Case fatality rate	Incidence	Mortality	Case fatality rate	Incidence	Mortality	Case fatality rate
Gini index	0·14*	0·17*	0·17*	0·16*	0·09*	0·09*	0·14*	0·10*	0·10*	0·11*	0·10*	0·10*	0·10*	0·17*	0·17*
Social Vulnerability Index (SVI)	0·05*	0·09*	0·08*	0·03	0·00	0·00	-0·02	0·00	0·00	-0·04*	-0·01	-0·01	0·00	0·08*	0·08*
SVI infrastructure	0·13*	0·14*	0·14*	0·11*	0·07*	0·07*	0·08*	0·07*	0·07*	0·06*	0·08*	0·09*	0·08*	0·14*	0·14*
SVI human capital	0·03	0·06*	0·05*	0·01	-0·03	-0·03	-0·04*	-0·03	-0·03	-0·06*	-0·04*	-0·04*	-0·02	0·06*	0·06*
SVI work and income	-0·03	0·03	0·02	-0·05*	-0·05*	-0·05*	-0·09*	-0·05*	-0·05*	-0·12*	-0·08*	-0·08*	-0·07*	0·01	0·01
Municipal human development index (MHDI)	0·05*	0·01	0·01	0·07*	0·07*	0·07*	0·12*	0·08*	0·08*	0·15*	0·11*	0·11*	0·09*	0·02	0·02
MHDI longevity	0·01	-0·03	-0·03	0·03	0·03	0·03	0·07*	0·04*	0·04*	0·09*	0·06*	0·06*	0·04*	-0·02	-0·02
MHDI education	0·06*	0·03	0·04*	0·08*	0·08*	0·08*	0·12*	0·08*	0·08*	0·14*	0·11*	0·11*	0·09*	0·04*	0·04*
MDHI income	0·06*	0·00*	0·00	0·08*	0·07*	0·07*	0·12*	0·08*	0·08*	0·15*	0·10*	0·10*	0·10*	0·02	0·02
People with low income (%)	0·00	0·05*	0·05*	-0·02	-0·03	-0·03	-0·07*	-0·04*	-0·04*	-0·10*	-0·06*	-0·06*	-0·05*	0·04*	0·04*
Unemployment (%)	0·18*	0·17*	0·17*	0·16*	0·09*	0·09*	0·13*	0·07*	0·08*	0·09*	0·11*	0·11*	0·12*	0·17*	0·17*
Households with inadequate water supply and sewage services (%)	-0·08*	0·09*	0·09*	-0·07*	-0·01	-0·01	-0·02	0·00	0·00	-0·01	-0·01	-0·01	-0·04*	0·10*	0·10*
Households with inadequate waste collection service (%)	-0·04*	0·08*	0·08*	-0·03	0·00	0·00	-0·02	0·00	0·00	0·00	0·00	-0·01	-0·02	0·08*	0·08*
Illiteracy people (%)	-0·06*	-0·02	-0·02	-0·09*	-0·07*	-0·07*	-0·14*	-0·08*	-0·08*	-0·16*	-0·12*	-0·12*	-0·10*	-0·03*	-0·03*
Hospital beds per 100,000 population	0·10*	0·07*	0·07*	0·11*	0·04*	0·04*	0·11*	0·05*	0·05*	0·15*	0·07*	0·07*	0·14*	0·13*	0·13*
Pre-existing Intensive Care Unit beds per 100,000 population	0·21*	0·26*	0·26*	0·28*	0·27*	0·27*	0·28*	0·26*	0·27*	0·21*	0·33*	0·34*	0·21*	0·28*	0·28*
Outpatient clinics per 100,000 population	-0·02	-0·03*	-0·03	-0·02	-0·01	-0·01	0·01	-0·01	-0·01	-0·04*	-0·01	-0·01	-0·02	-0·04*	-0·04*
Family Health Strategy coverage (%)	-0·25*	-0·18*	-0·18*	-0·28*	-0·14*	-0·14*	-0·30*	-0·16*	-0·16*	-0·30*	-0·23*	-0·24*	-0·22*	-0·23*	-0·23*
New Intensive Care Unit beds per 100,000 population	0·06*	0·11*	0·11*	0·09*	0·17*	0·17*	0·08*	0·12*	0·12*	0·07*	0·14*	0·14*	0·21*	0·28*	0·28*
Physicians’ coverage (%)	0·13*	0·10*	0·10*	0·14*	0·11*	0·11*	0·16*	0·10*	0·10*	0·18*	0·14*	0·14*	0·13*	0·11*	0·11*
Nurses’ coverage (%)	0·07*	0·07*	0·07*	0·09*	0·08*	0·08*	0·10*	0·09*	0·09*	0·13*	0·10*	0·10*	0·09*	0·07*	0·07*

⁎ *P*-value < 0·05.

Table 2 shows the Beta regression for the principal components of the socio-economic variables’ matrix by age group. High COVID-19 incidence for all age groups was identified in municipalities with a lower percentage of people living on low income, with a lower percentage of unemployed people, lower SVI scores for human capital and work and income, a lower percentage of illiterate people, and a higher MHDI. Municipalities with the lowest health service coverage (lower rate of outpatient clinics, Family Program health teams and paediatric hospital beds), higher social inequality, higher SVI scores in infrastructure, higher percentage of households with inadequate water supply, sewage and waste collection services had also higher COVID-19 incidence for all age groups.

**Table 2:** Beta regression model for the incidence, mortality and case fatality rates due to COVID-19 in children and adolescents according to paediatric age group in Brazil from March 2020 to October 2021.

Variables	0–4 years			5–9 years			10–14 years			15–19 years			0–19 years		
	Estimate	95%CI*	*P*-value	Estimate	95%CI*	*P*-value	Estimate	95%CI*	*P*-value	Estimate	95%CI*	*P*-value	Estimate	95%CI*	*P*-value
Incidence rate															
Intercept	-7·27	-7·33 to -7·21	0·000	-8·50	-8·57 to -8·43	0·000	-8·40	-8·47 to -8·33	0·000	-7·54	-7·60 to -7·47	0·000	-7·84	-7·90 to -7·79	<0·001
PCA⁎⁎ 1	0·04	0·02 to 0·07	0·001	0·04	0·02 to 0·07	0·002	0·06	0·04 to 0·09	0·000	0·10	0·08 to 0·13	0·000	0·07	0·04 to 0·09	<0·001
PCA⁎⁎ 2	-0·16	-0·18 to -0·13	0·000	-0·12	-0·15 to -0·10	0·000	-0·12	-0·15 to -0·10	0·000	-0·15	-0·18 to -0·13	0·000	-0·17	-0·19 to -0·14	<0·001
PCA⁎⁎ 3	0·20	0·17 to 0·22	0·000	0·13	0·10 to 0·15	0·000	0·12	0·09 to 0·14	0·000	0·18	0·15 to 0·20	0·000	0·27	0·24 to 0·29	<0·001
PCA⁎⁎ 4	0·09	0·06 to 0·11	0·000	0·06	0·04 to 0·09	0·000	0·06	0·04 to 0·09	0·000	0·08	0·05 to 0·11	0·000	0·11	0·08 to 0·13	<0·001
PCA⁎⁎ 5	0·06	0·04 to 0·09	0·000	0·03	0·01 to 0·06	0·009	0·03	0·00 to 0·05	0·038	0·03	0·00 to 0·05	0·047	0·05	0·02 to 0·07	<0·001
Mortality rate															
Intercept	-9·62	-9·68 to -9·57	0·000	-11·22	-11·27 to -11·17	0·000	-10·74	-10·8 to -10·69	0·000	-9·80	-9·86 to -9·75	0·000	-10·22	-10·31 to -10·13	<0·001
PCA⁎⁎ 1	-0·01	-0·04 to 0·01	0·263	0·00	-0·02 to 0·03	0·743	0·01	-0·01 to 0·04	0·419	0·02	0·00 to 0·05	0·068	0·01	-0·02 to 0·03	0·688
PCA⁎⁎ 2	-0·07	-0·09 to -0·04	0·000	-0·04	-0·06 to -0·01	0·005	-0·03	-0·05 to 0·00	0·025	-0·06	-0·08 to -0·03	0·000	-0·11	-0·14 to -0·09	<0·001
PCA⁎⁎ 3	0·06	0·03 to 0·08	0·000	0·02	0·00 to 0·05	0·113	0·02	0·00 to 0·05	0·076	0·05	0·03 to 0·08	0·000	0·10	0·08 to 0·13	<0·001
PCA⁎⁎ 4	0·04	0·01 to 0·06	0·006	0·01	-0·02 to 0·03	0·532	0·01	-0·01 to 0·04	0·270	0·03	0·00 to 0·05	0·029	0·06	0·03 to 0·09	<0·001
PCA⁎⁎ 5	0·03	0·00 to 0·05	0·039	0·01	-0·01 to 0·04	0·399	0·00	-0·02 to 0·03	0·813	0·01	-0·01 to 0·04	0·275	0·04	0·01 to 0·07	0.002
Case fatality rate															
Intercept	-1·81	-1·84 to -1·79	0·000	-2·13	-2·15 to -2·11	0·000	-2·03	-2·05 to -2·01	0·000	-1·79	-1·81 to -1·76	0·000	-1·66	-1·68 to -1·63	<0·001
PCA⁎⁎ 1	-0·04	-0·06 to -0·02	0·000	-0·01	-0·02 to 0·01	0·310	0·00	-0·01 to 0·02	0·699	0·01	-0·02 to 0·03	0·593	-0·05	-0·07 to -0·03	<0·001
PCA⁎⁎ 2	-0·04	-0·06 to -0·02	0·000	-0·04	-0·06 to -0·03	0·000	-0·03	-0·05 to -0·01	0·002	-0·04	-0·06 to -0·02	0·000	-0·04	-0·06 to -0·02	0·001
PCA⁎⁎ 3	0·05	0·03 to 0·07	0·000	0·02	0·01 to 0·04	0·004	0·04	0·02 to 0·06	0·000	0·07	0·05 to 0·09	0·000	0·06	0·04 to 0·09	<0·001
PCA⁎⁎ 4	0·04	0·02 to 0·06	0·000	0·01	-0·01 to 0·02	0·418	0·02	0·00 to 0·04	0·049	0·03	0·01 to 0·05	0·003	0·04	0·02 to 0·06	<0·001
PCA⁎⁎ 5	0·02	0·00 to 0·04	0·117	0·02	0·00 to 0·03	0·057	0·00	-0·02 to 0·02	0·839	0·01	-0·01 to 0·04	0·190	0·01	-0·01 to 0·04	0·237

⁎ 95%CI: Confidence interval 95%.

⁎⁎ PCA: Principal Component Analysis.

The relationship between child and adolescent COVID-19 mortality rate and socio-economic indicators is shown in Table 2. Municipalities with low pre-existing ICU rates, low rates of new ICUs in response to COVID-19, and low average physicians’ and nurses’ coverage (lower than the average) had lower mortality rates for all age groups. Conversely, municipalities with the low rate of outpatient clinics per 100,000 inhabitants and low percentage of family health teams, as well as in those with higher social inequality (higher Gini index), higher social vulnerability in relation to infrastructure (high SVI scores in infrastructure), higher percentage of households with inadequate piped water and sewage collection, higher percentage of households with inadequate waste collection had the highest mortality rates for the age groups 0–4 years old and 15–19 years old. The relationship between municipalities with lower hospital bed rates and higher mortality rates was observed only for the 0–4 age group.

Table 2 also shows the relationship between CFR and PCAs. Lower case fatality rates were related to better socioeconomic indicators and better MHDI scores for the children aged 0–4 years. Municipalities with low pre-existing ICU rates, low rates of new ICUs in response to COVID-19, and low average physicians’ and nurses’ coverage (lower than the average) had also lower CFR for all age groups. However, municipalities with the low rate of outpatient clinics per 100,000 inhabitants and low percentage of family health teams, as well as in those with higher social inequality, higher social vulnerability in relation to infrastructure, higher percentage of households with inadequate piped water and sewage collection, higher percentage of households with inadequate waste collection had the highest case fatality rates.

## Discussion

These population-based analyses of COVID-19 incidence and deaths confirm the burden of disease in the paediatric population in Brazil, its heterogeneous geographic distribution, and well-defined spatial clusters in the North and Northeast regions. Our findings describe a higher mortality rate in municipalities with higher social inequality and vulnerability and worse social and economic indicators.

Although COVID-19 cases and deaths among children and adolescents represent a small fraction of the burden of disease worldwide, Brazil has recorded a high number of cases (*n* = 33,991) and deaths (*n* = 2,424), which concentrate in areas with large social and health inequity. Their geographical distribution resembles the distribution of cases among the general^18^^,^^26^^,^^27^ and obstetric population,^28^ which also have higher incidence and mortality in municipalities with marked socioeconomic vulnerabilities and health inequities, and are mainly located in the North and Northeast regions.

In Brazil, the COVID-19 pandemic has exacerbated the municipalities predating social, economic, and infrastructural inequalities, especially in historically neglected regions. These dissimilarities contributed to the spread of SARS-CoV-2 in the paediatric population living in socially vulnerable areas, resembling similar vulnerabilities reported from adults,^29^ and increasing the risk of illness and death.^6^^,^^30^ The limited and precarious child health care network in these areas is linked to unfavourable outcomes, especially among the youngest.^7^^,^^13^^,^^31^

In anticipation of the spread of SARS-CoV-2, approximately 60% of Brazilian municipalities expanded their health services capacity with temporary health facilities for the care of adults and children with like-flu symptoms, expanding their capacity for testing of COVID-19 presumptive cases. These actions were added to the already existing health network, which improved people's access to SARS-CoV-2 testing, particularly in the municipalities with better socioeconomic indicators. The more health facilities providing care and testing for COVID-19, the higher the probability of detecting incident cases, which may explain the high incidence seen in municipalities with high MHDI, high pre-existing and new ICUs and high physicians’ and nurses’ coverage in our analysis.

Paradoxically, our analyses showed that municipalities with higher pre-existing and new ICU rates and higher average physicians’ and nurses’ coverage had higher mortality and CFR. Although apparently counter-intuitive, higher pre-existing and new ICU rates and physicians’ and nurses’ coverage does not necessarily indicate a municipality has a well-established health network with equity of access. Although metropolitan cities often have higher ICU beds availability and higher numbers of health professionals per inhabitant, these cities also have an unequal social and geographical distribution of resources, resulting in unequal access to services, with people living in deprived areas having less opportunity for health care.^6^^,^^30^ Overall, however, in view of the set of socioeconomic and social vulnerability indicators, our findings showed a relationship between social inequities and vulnerabilities and the burden of COVID-19 in the paediatric population.

New hospital and ICU beds were opened in approximately 13% of Brazilian municipalities increasing the number of hospital and ICU beds.^18^^,^^28^ Despite this, Brazil faced difficulties in re-organizing the health system to cope with the pandemic,^18^^,^^32^ as the availability of beds is uneven across the municipalities and regions. Historically, hospital and ICU beds have concentrated in larger municipalities with higher *per capita* income and heterogeneity in their distribution between the public and private sector.^33^

The Southeast region, which has the best socioeconomic indicators, has 4·1 paediatric ICU bed per 10,000 children aged 0–14 years. However, there are only 2·7 paediatric ICU beds per 10,000 children in the public sector and 7·1 paediatric ICU beds/10,000 children in the private sector. In contrast, the Northeast and North regions, which have the worst socioeconomic and health indicators, have the lowest proportions of paediatric ICU beds. The Northeast region has an average of 1·6 beds per 10,000 children, of which the public and private sectors have 1·1 and 6·0 paediatric ICU beds per 10,000 children, respectively, while the North has the lowest number of beds with only 1.0 and 8.6 UCI beds/10,000 children in the public and private sectors, respectively.^34^ These disparities reflect the regions different access to hospital health care for children.

Describing geographic areas with the highest risk of COVID-19 mortality in children and adolescents would assist policymakers in the allocation of resources to mitigate the burden of SARS-CoV-2, as to expand the healthcare network to the areas in greater need. The identification of spatial clusters at high risk of death is useful to prioritise vaccine doses and intensification of immunisations through specific campaigns and allocation of staff and resources. Since 2021, many countries have given emergency use authorization for mRNA vaccines for adolescents (aged 12–17 years);^35^ and trials have demonstrated the safety and efficacy of the vaccines for younger children aged 5 to 11 years,^35^, ^36^, ^37^, ^38^ with further vaccines undergoing trials in children as young as 6 months.

This study assessed a large sample size of children and adolescents with COVID-19 and how social determinants of health are associated with an increased risk of death in a country with marked social inequalities. Nevertheless, the results presented here need to be interpreted according to the study limitations. The study has primarily captured data from symptomatic children and adolescents who attended the health services and were tested for SARS-CoV-2. Consequently, children and adolescents who were not seen in the health services would have been undetected. Given that access to health services was not homogeneous, missing and undetected cases would be more likely to occur in disadvantaged populations, which may under-represent those who live in the areas with poorer conditions. Consequently, undetected cases in poor communities could have influenced the detection and CFR, as higher underreporting is expected in areas with poor access to health services. Social, demographic and economic data were also extracted from the 2010 National Census and are becoming dated. However, the Brazilian federal government cancelled the 2020 census and the data we used is the most recent data available. Moreover, the Ministry of Health has not updated the SIVEP-Gripe database since October 2021, and cases due to the Omicron strain have not been analysed. As the Omicron strain has higher infectivity, but lower mortality, the access to services may have also changed. Lastly, secondary data and ecological studies are unsuitable to establish disease causality, and therefore, our analyses only provide evidence of statistically significant relationships between COVID-19, poverty and social inequalities.

Although COVID-19 cases and deaths among children and adolescents occurred in all regions of Brazil, the main clusters of high mortality occurred in municipalities in the North and Northeast, which are the regions with the worst socioeconomic indicators and greatest health disparities.

Our findings confirm the relationship between social inequities and vulnerabilities and the burden of COVID-19 in the paediatric population. Addressing these relationships would require social and infrastructure policies to reduce the disparities across the regions. Identifying geographical areas with the highest risk of death can be useful for implementing contingency measures to reduce the burden of the pandemic and prioritise areas for the intensification of mass vaccination of children and adolescents. COVID-19 vaccines are currently recommended in Brazil since January 2022 for individuals over 5 years of age and these areas should be prioritised. Our findings reinforce the need for immunisation against COVID-19 of younger children (under 4 years old), once the safety of the vaccines for this population has been proven, since high mortality rates have been identified in Brazil. Continued monitoring of cases and deaths from COVID-19 in children is needed even after the adoption of vaccination to identify potential changes in the dynamics of COVID-19 pandemic.

## Contributors

VSS: conceptualisation, methodology, project administration, supervision, data curation, interpretation, and writing. TSS: conceptualisation, methodology, data collection, and writing original draft. JRSS: data collection, formal data analysis, figures, and interpretation. AICA, MARRS, SCFV, ASAL, PRMF and RQG: literature search, data interpretation, and writing. LEC: data interpretation, review, and writing. All authors discussed the results and contributed to the final manuscript.

## Data sharing statement

SIVEP-Gripe dataset and all other databases used in this study are publicly available. Our analysis code is available upon request to the corresponding author.

## Editor note

The Lancet Group takes a neutral position with respect to territorial claims in published maps and institutional affiliations.

## Declaration of interests

The authors declare that there is no conflict of interest.

## Appendix Supplementary materials

Image, application 1

Image, application 2
