# Supplementary material for: Spatial clusters, social determinants of health and risk of COVID-19 mortality in Brazilian children and adolescents: A nationwide population-based ecological study
Source: Lancet Reg Health Am. 2022 Jun 29;13:100311. doi: 10.1016/j.lana.2022.100311 (PMC9242540; doi:10.1016/j.lana.2022.100311)
Supplement: Supplementary file 1 [file mmc1.docx]

**Appendix**

**Spatial clusters, social determinants of health and risk of COVID-19 mortality in Brazilian children and adolescents: a nationwide population-based ecological study**

**Supplementary material (S.1)
SIVEP-Gripe (The Influenza Epidemiological Surveillance Information System)**

SIVEP-Gripe is a nationwide database established by Brazilian Ministry of Health in 2009 for the surveillance of acute respiratory distress syndromes (ARDS). With the emergence of the COVID-19 pandemic in Brazil, SIVEP-Gripe was redesigned in March 2020, allowing the notification of ARDS cases due to COVID-19. SIVEP-Gripe has been the primary source of information on COVID-19 related hospital admissions and deaths in Brazil. COVID-19 case notification is mandatory, and records are stored in the computerized SIVEP-Gripe database, and SIVEP-Gripe receives notification of patients admitted to both private and public hospitals.

To obtain the number of cases and maternal deaths due to COVID-19 according to the municipality of residence, and to minimize inconsistencies, we tracked the following procedure:

1. We filtered out in “CLASSI_FIN” variable only cases of ARDS due to COVID-19 confirmed by RT-PCR. For this purpose, we only consider cases reported as numeral 5.
2. We used the variables “NU_IDADE_N” and “TP_IDADE” to identify age groups. When “TP_IDADE” = 1, “NU_IDADE” is described in days, when “TP_IDADE” = 2, “NU_IDADE_N” is described in months, and when “TP_IDADE” = 3, “NU_IDADE_N” is described in years. Thus, we only consider cases reported where 0 ≤ “NU_IDADE_N” ≤ 30 for “TP_IDADE” = 1, 0 ≤ “NU_IDADE_N” ≤ 12 for “TP_IDADE” = 2 and 0 ≤ “NU_IDADE_N” ≤ 19 for “ TP_IDADE” = 3.
3. For the special analysis, we used the patient's municipality of residence as a reference, identified by the variable “CO_MUN_RES”.

**Supplementary Table 1. Definition and source of demographic, socioeconomic, and healthcare indicators used in the study.**

| **Variable** | **Definition** | **Data source** | **Period** |
| --- | --- | --- | --- |
| **Demographic** | | | |
| Estimated total population of the municipality | Represents the estimated population of each municipality | DATASUS – Resident-population- estimates from TCU | 2021 |
| **Socioeconomic** | | | |
| Gini index | This indicator estimates or grades of inequality of a country | Brazilian Institute of Geography and Statistics - Demographic Census | 2010 |
| Social Vulnerability Index (SVI) | This index estimates the degree of vulnerability and social exclusion of a population | Brazilian Institute of Geography and Statistics - Demographic Census | 2010 |
| SVI infrastructure | Represents one of the dimensions of SVI, estimates the infrastructure of the territory | Institute of Applied Economic Research | 2010 |
| SVI human capital | Represents one of the dimensions of the IVS, estimates the human capital of the households belonging to the territory | Institute of Applied Economic Research | 2010 |
| SVI work and income | Represents one of the dimensions of IVS, estimating income or access to work and the form of insertion | Institute of Applied Economic Research | 2010 |
| Municipal human development index (MHDI) | Measures and evaluates the development of a country according to its social and economic aspects | Brazilian Institute of Geography and Statistics - Demographic Census | 2010 |
| MHDI longevity | Represents one of the dimensions of the Municipal Human Development Index, estimates longevity in the municipality | Brazilian Institute of Geography and Statistics - Demographic Census | 2010 |
| MHDI education | It represents one of the dimensions of the Municipal Human Development Index, estimates education in the municipality | Brazilian Institute of Geography and Statistics - Demographic Census | 2010 |
| MDHI income | Represents one of the dimensions of the Municipal Human Development Index, estimates the municipal income | Brazilian Institute of Geography and Statistics - Demographic Census | 2010 |
| People with low income (%) | Estimates the percentage of people living with low income in Brazilian municipalities | Brazilian Institute of Geography and Statistics - Demographic Census | 2010 |
| Unemployment (%) | Estimate the percentage of unemployed in Brazilian municipalities | Brazilian Institute of Geography and Statistics - Demographic Census | 2010 |
| Households with inadequate water supply and sewage services (%) | Estimates the percentage of households with inadequate sanitation services | Brazilian Institute of Geography and Statistics - Demographic Census | 2010 |
| Households with inadequate rubbish collection service (%) | Measures the percentage of households with inadequate rubbish collection service (%) | Brazilian Institute of Geography and Statistics - Demographic Census | 2010 |
| Illiteracy people (%) | Measures the percentage of illiterate people by municipality | Brazilian Institute of Geography and Statistics - Demographic Census | 2010 |
| **Healthcare** | | | |
| Hospital beds per 100,000 population | Measures the percentage of hospital beds per municipality | Ministry of Health's National Registry of Health Establishments (CNES) | March 2020 to October 2021 |
| Pre-existing Intensive Care Unit beds per 100,000 population | Measures the percentage of pre-existing ICU beds before the COVID 19 pandemic | Ministry of Health's National Registry of Health Establishments (CNES) | March 2020 to October 2021 |
| Outpatient clinics per 100,000 population | Measures the percentage of municipal outpatient clinics | Ministry of Health's National Registry of Health Establishments (CNES) | March 2020 to October 2021 |
| Family Health Strategy coverage (%) | Measures the coverage of the Family Health Strategy by municipality | Ministry of Health's National Registry of Health Establishments (CNES) | March 2020 to October 2021 |
| New Intensive Care Unit beds per 100,000 population | Measures the percentage of new ICU beds created during the COVID 19 pandemic | Ministry of Health's National Registry of Health Establishments (CNES) | March 2020 to October 2021 |
| Physicians’ coverage (%) | Measures the coverage of doctors by municipality | Ministry of Health's National Registry of Health Establishments (CNES) | March 2020 to October 2021 |
| Nurses’ coverage (%) | Measures the coverage of nurses by municipality | Ministry of Health's National Registry of Health Establishments (CNES) | March 2020 to October 2021 |

**Supplementary Table 2. Principal component matrix using varimax rotation method with Kaiser normalization.**

| **Variable** | **PCA 1** | **PCA 2** | **PCA 3** | **PCA 4** | **PCA 5** |
| --- | --- | --- | --- | --- | --- |
| Gini index |  |  |  | 0·44 |  |
| Low income | -0·90 |  |  |  |  |
| Unemployment (%) | -0·47 |  |  |  |  |
| Social Vulnerability Index (SVI) | -0·78 |  |  |  |  |
| SVI infrastructure |  |  |  | 0·83 |  |
| SVI human capital | -0·86 |  |  |  |  |
| SVI work and income | -0·89 |  |  |  |  |
| Water and sanitation (%) |  |  |  | -0·62 |  |
| Waste collection |  |  |  | -0·77 |  |
| Illiteracy (%) | -0·88 |  |  |  |  |
| MHDI | 0·92 |  |  |  |  |
| MHDI longevity | 0·80 |  |  |  |  |
| MHDI education | 0·84 |  |  |  |  |
| MDHI income | 0·90 |  |  |  |  |
| Hospital beds |  |  |  |  | -0·88 |
| ICU beds (pre-existing) |  | -0·65 |  |  |  |
| Outpatient clinics |  |  | -0·79 |  |  |
| Family Health team coverage |  |  | -0·72 |  |  |
| New ICU beds |  | -0·36 |  |  |  |
| Physicians’ coverage (%) |  | -0·71 |  |  |  |
| Nurse’ coverage (%) |  | -0·70 |  |  |  |

**
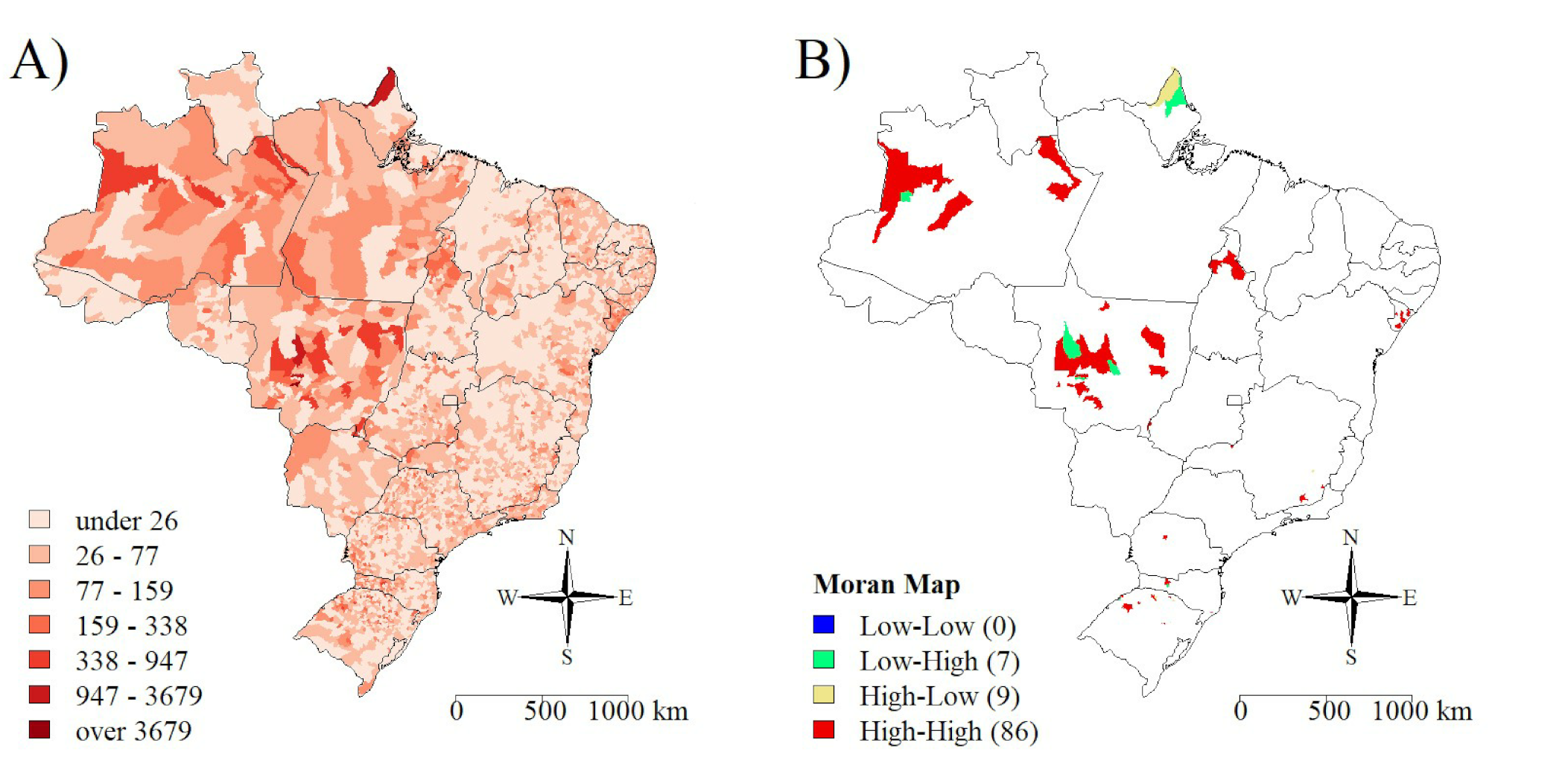
**

**Supplementary Figure 1. Spatial distribution of A) the COVID 19 incidence rate per 100,000 children and adolescents aged 0-19 years and B) Moran Map for Children aged 0-19 years, in Brazil from March 2020 to October 2021.**

**Supplementary Table 3. Number of municipalities by Region/State by age group according to COVID-19 incidence rate stratum.**

| **Age group/Region/State** | **Stratum** | | |
| --- | --- | --- | --- |
|  | **High-High** | **High-Low** | **Low-High** |
| **0-4 years old** | **No. municipalities** | **No. municipalities** | **No. municipalities** |
| **Brazil** | **157** | **33** | **27** |
| **North** | **33** | **4** | **2** |
| Acre | 0 | 0 | 0 |
| Amapá | 0 | 1 | 1 |
| Amazonas | 13 | 1 | 0 |
| Pará | 2 | 0 | 0 |
| Rondônia | 0 | 0 | 0 |
| Roraima | 0 | 0 | 0 |
| Tocantins | 18 | 2 | 1 |
| **Northeast** | **63** | **4** | **2** |
| Alagoas | 11 | 0 | 1 |
| Bahia | 1 | 1 | 0 |
| Ceará | 9 | 0 | 0 |
| Maranhão | 0 | 0 | 0 |
| Paraíba | 1 | 2 | 0 |
| Pernambuco | 0 | 0 | 0 |
| Piauí | 0 | 0 | 0 |
| Rio Grande do Norte | 2 | 1 | 0 |
| Sergipe | 39 | 0 | 1 |
| **Central-West** | **19** | **3** | **3** |
| Goiás | 2 | 2 | 1 |
| Mato Grosso | 17 | 1 | 2 |
| Mato Grosso do Sul | 0 | 0 | 0 |
| **Southeast** | **20** | **7** | **7** |
| Espírito Santo | 0 | 0 | 0 |
| Minas Gerais | 15 | 6 | 7 |
| Rio de Janeiro | 0 | 0 | 0 |
| São Paulo | 5 | 1 | 0 |
| **South** | **22** | **15** | **13** |
| Paraná | 7 | 2 | 1 |
| Rio Grande do Sul | 8 | 10 | 9 |
| Santa Catarina | 7 | 3 | 3 |
| **5-9 years old** |  |  |  |
| **Brazil** | **72** | **18** | **10** |
| **North** | **16** | **1** | **0** |
| Acre | 0 | 0 | 0 |
| Amapá | 1 | 1 | 0 |
| Amazonas | 6 | 0 | 0 |
| Pará | 1 | 0 | 0 |
| Rondônia | 0 | 0 | 0 |
| Roraima | 0 | 0 | 0 |
| Tocantins | 8 | 0 | 0 |
| **Northeast** | **27** | **4** | **1** |
| Alagoas | 6 | 0 | 0 |
| Bahia | 4 | 1 | 0 |
| Ceará | 1 | 1 | 0 |
| Maranhão | 0 | 0 | 0 |
| Paraíba | 0 | 0 | 0 |
| Pernambuco | 0 | 0 | 0 |
| Piauí | 0 | 0 | 0 |
| Rio Grande do Norte | 0 | 1 | 0 |
| Sergipe | 16 | 1 | 1 |
| **Central-West** | **14** | **2** | **3** |
| Goiás | 1 | 0 | 0 |
| Mato Grosso | 13 | 2 | 3 |
| Mato Grosso do Sul | 0 | 0 | 0 |
| **Southeast** | **7** | **3** | **6** |
| Espírito Santo | 0 | 0 | 0 |
| Minas Gerais | 2 | 1 | 0 |
| Rio de Janeiro | 1 | 0 | 0 |
| São Paulo | 4 | 2 | 6 |
| **South** | **8** | **8** | **0** |
| Paraná | 0 | 3 | 0 |
| Rio Grande do Sul | 4 | 3 | 0 |
| Santa Catarina | 4 | 2 | 0 |
| **10-14 years old** |  |  |  |
| **Brazil** | **38** | **11** | **5** |
| **North** | **3** | **0** | **0** |
| Acre | 0 | 0 | 0 |
| Amapá | 0 | 0 | 0 |
| Amazonas | 2 | 0 | 0 |
| Pará | 1 | 0 | 0 |
| Rondônia | 0 | 0 | 0 |
| Roraima | 0 | 0 | 0 |
| Tocantins | 0 | 0 | 0 |
| **Northeast** | **5** | **0** | **0** |
| Alagoas | 0 | 0 | 0 |
| Bahia | 0 | 0 | 0 |
| Ceará | 0 | 0 | 0 |
| Maranhão | 0 | 0 | 0 |
| Paraíba | 0 | 0 | 0 |
| Pernambuco | 0 | 0 | 0 |
| Piauí | 0 | 0 | 0 |
| Rio Grande do Norte | 0 | 0 | 0 |
| Sergipe | 5 | 0 | 0 |
| **Central-West** | **14** | **1** | **2** |
| Goiás | 1 | 0 | 0 |
| Mato Grosso | 13 | 1 | 2 |
| Mato Grosso do Sul | 0 | 0 | 0 |
| **Southeast** | **4** | **4** | **0** |
| Espírito Santo | 0 | 0 | 0 |
| Minas Gerais | 2 | 3 | 0 |
| Rio de Janeiro | 0 | 0 | 0 |
| São Paulo | 2 | 1 | 0 |
| **South** | **12** | **6** | **3** |
| Paraná | 2 | 1 | 0 |
| Rio Grande do Sul | 7 | 3 | 3 |
| Santa Catarina | 3 | 2 | 0 |
| **15-19 years old** |  |  |  |
| **Brazil** | **52** | **6** | **6** |
| **North** | **11** | **2** | **1** |
| Acre | 0 | 0 | 0 |
| Amapá | 0 | 1 | 0 |
| Amazonas | 7 | 0 | 1 |
| Pará | 0 | 0 | 0 |
| Rondônia | 0 | 0 | 0 |
| Roraima | 0 | 0 | 0 |
| Tocantins | 0 | 0 | 0 |
| **Northeast** | **1** | **1** | **0** |
| Alagoas | 0 | 0 | 0 |
| Bahia | 0 | 0 | 0 |
| Ceará | 0 | 0 | 0 |
| Maranhão | 0 | 0 | 0 |
| Paraíba | 1 | 1 | 0 |
| Pernambuco | 0 | 0 | 0 |
| Piauí | 0 | 0 | 0 |
| Rio Grande do Norte | 0 | 0 | 0 |
| Sergipe | 0 | 0 | 0 |
| **Central-West** | **15** | **0** | **1** |
| Goiás | 3 | 0 | 0 |
| Mato Grosso | 12 | 0 | 1 |
| Mato Grosso do Sul | 0 | 0 | 0 |
| **Southeast** | **1** | **0** | **0** |
| Espírito Santo | 0 | 0 | 0 |
| Minas Gerais | 1 | 0 | 0 |
| Rio de Janeiro | 0 | 0 | 0 |
| São Paulo | 0 | 0 | 0 |
| **South** | **24** | **3** | **4** |
| Paraná | 4 | 0 | 0 |
| Rio Grande do Sul | 13 | 3 | 4 |
| Santa Catarina | 7 | 0 | 0 |

**
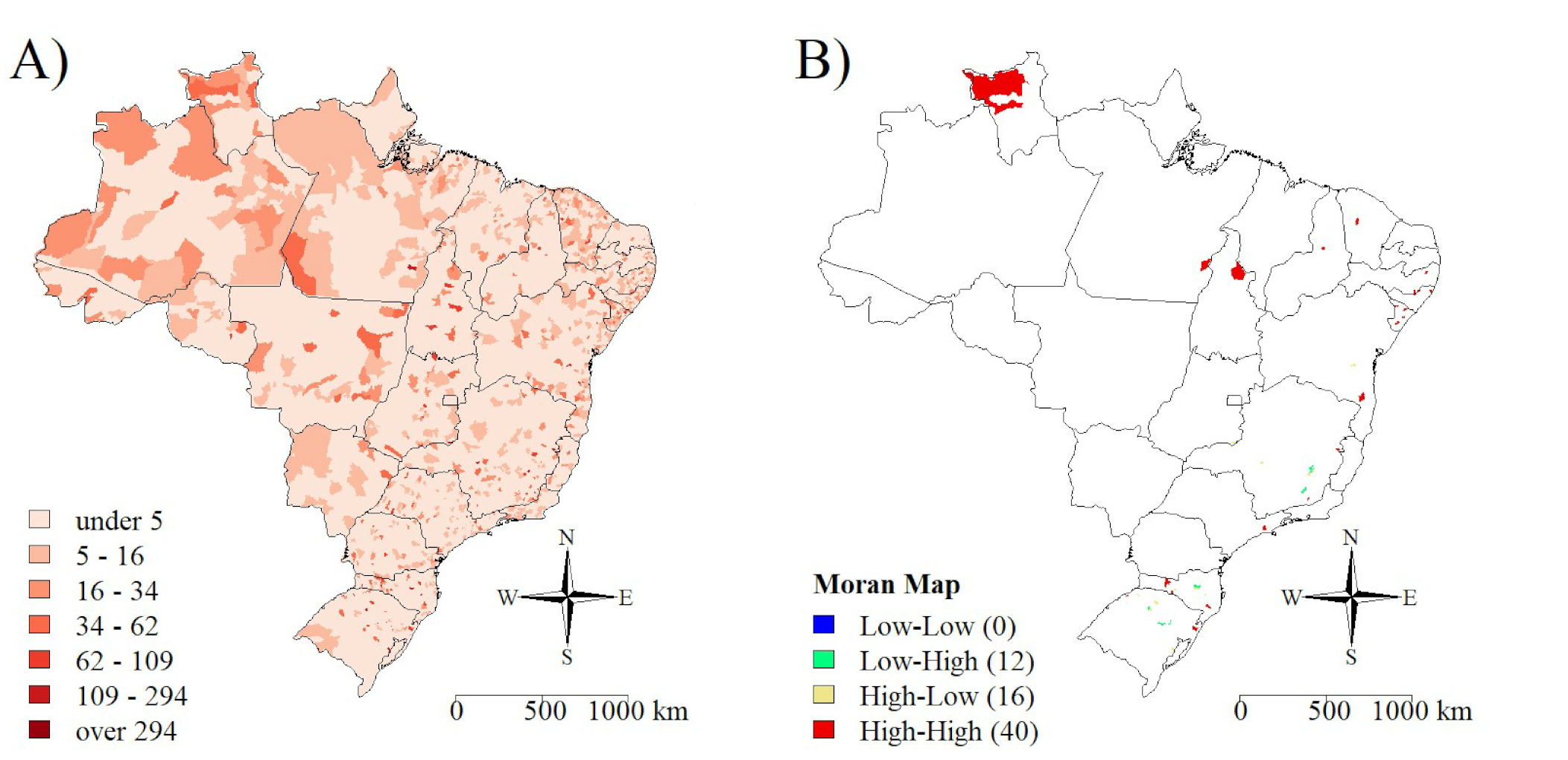
**

**Supplementary Figure 2. Spatial distribution of A) the COVID 19 mortality rate per 100,000 children and adolescents aged 0-19 years and B) Moran Map for Children aged 0-19 years, in Brazil from March 2020 to October 2021.**

**Supplementary Table 4. Number of municipalities by Region/State by age group according to COVID-19 mortality rate stratum.**

| **Age group/Region/State** | **Stratum** | | |
| --- | --- | --- | --- |
|  | **High-High** | **High-Low** | **Low-High** |
| **0-4 years old** | **No. municipalities** | **No. municipalities** | **No. municipalities** |
| **Brazil** | **19** | **12** | **5** |
| **North** | **2** | **0** | **0** |
| Acre | 0 | 0 | 0 |
| Amapá | 0 | 0 | 0 |
| Amazonas | 0 | 0 | 0 |
| Pará | 2 | 0 | 0 |
| Rondônia | 0 | 0 | 0 |
| Roraima | 0 | 0 | 0 |
| Tocantins | 0 | 0 | 0 |
| **Northeast** | **13** | **2** | **0** |
| Alagoas | 1 | 0 | 0 |
| Bahia | 0 | 1 | 0 |
| Ceará | 6 | 0 | 0 |
| Maranhão | 0 | 0 | 0 |
| Paraíba | 0 | 0 | 0 |
| Pernambuco | 2 | 0 | 0 |
| Piauí | 0 | 0 | 0 |
| Rio Grande do Norte | 0 | 1 | 0 |
| Sergipe | 4 | 0 | 0 |
| **Central-West** | **0** | **1** | **0** |
| Goiás | 0 | 1 | 0 |
| Mato Grosso | 0 | 0 | 0 |
| Mato Grosso do Sul | 0 | 0 | 0 |
| **Southeast** | **2** | **7** | **5** |
| Espírito Santo | 0 | 0 | 0 |
| Minas Gerais | 2 | 7 | 5 |
| Rio de Janeiro | 0 | 0 | 0 |
| São Paulo | 0 | 0 | 0 |
| **South** | **2** | **2** | **0** |
| Paraná | 0 | 0 | 0 |
| Rio Grande do Sul | 0 | 2 | 0 |
| Santa Catarina | 2 | 0 | 0 |
| **5-9 years old** |  |  |  |
| **Brazil** | **3** | **3** | **2** |
| **North** | **0** | **0** | **0** |
| Acre | 0 | 0 | 0 |
| Amapá | 0 | 0 | 0 |
| Amazonas | 0 | 0 | 0 |
| Pará | 0 | 0 | 0 |
| Rondônia | 0 | 0 | 0 |
| Roraima | 0 | 0 | 0 |
| Tocantins | 0 | 0 | 0 |
| **Northeast** | **3** | **1** | **0** |
| Alagoas | 0 | 0 | 0 |
| Bahia | 0 | 0 | 0 |
| Ceará | 0 | 0 | 0 |
| Maranhão | 0 | 0 | 0 |
| Paraíba | 0 | 0 | 0 |
| Pernambuco | 0 | 0 | 0 |
| Piauí | 0 | 0 | 0 |
| Rio Grande do Norte | 0 | 0 | 0 |
| Sergipe | 3 | 1 | 0 |
| **Central-West** | **0** | **0** | **0** |
| Goiás | 0 | 0 | 0 |
| Mato Grosso | 0 | 0 | 0 |
| Mato Grosso do Sul | 0 | 0 | 0 |
| **Southeast** | 0 | 0 | 0 |
| Espírito Santo | 0 | 0 | 0 |
| Minas Gerais | 0 | 0 | 0 |
| Rio de Janeiro | 0 | 0 | 0 |
| São Paulo | 0 | 0 | 0 |
| **South** | **0** | **2** | **2** |
| Paraná | 0 | 0 | 0 |
| Rio Grande do Sul | 0 | 1 | 1 |
| Santa Catarina | 0 | 1 | 1 |
| **10-14 years old** |  |  |  |
| **Brazil** | **3** | **9** | **4** |
| **North** | **0** | **0** | **0** |
| Acre | 0 | 0 | 0 |
| Amapá | 0 | 0 | 0 |
| Amazonas | 0 | 0 | 0 |
| Pará | 0 | 0 | 0 |
| Rondônia | 0 | 0 | 0 |
| Roraima | 0 | 0 | 0 |
| Tocantins | 0 | 0 | 0 |
| **Northeast** | **0** | **2** | **0** |
| Alagoas | 0 | 0 | 0 |
| Bahia | 0 | 0 | 0 |
| Ceará | 0 | 1 | 0 |
| Maranhão | 0 | 0 | 0 |
| Paraíba | 0 | 0 | 0 |
| Pernambuco | 0 | 0 | 0 |
| Piauí | 0 | 1 | 0 |
| Rio Grande do Norte | 0 | 0 | 0 |
| Sergipe | 0 | 0 | 0 |
| **Central-West** | **0** | **0** | **0** |
| Goiás | 0 | 0 | 0 |
| Mato Grosso | 0 | 0 | 0 |
| Mato Grosso do Sul | 0 | 0 | 0 |
| **Southeast** | **2** | **3** | **0** |
| Espírito Santo | 0 | 0 | 0 |
| Minas Gerais | 0 | 1 | 0 |
| Rio de Janeiro | 0 | 0 | 0 |
| São Paulo | 2 | 2 | 0 |
| **South** | **1** | **4** | **4** |
| Paraná | 0 | 2 | 0 |
| Rio Grande do Sul | 0 | 1 | 0 |
| Santa Catarina | 1 | 1 | 4 |
| **15-19 years old** |  |  |  |
| **Brazil** | **7** | **8** | **5** |
| **North** | **0** | **1** | **0** |
| Acre | 0 | 0 | 0 |
| Amapá | 0 | 0 | 0 |
| Amazonas | 0 | 0 | 0 |
| Pará | 0 | 0 | 0 |
| Rondônia | 0 | 0 | 0 |
| Roraima | 0 | 0 | 0 |
| Tocantins | 0 | 1 | 0 |
| **Northeast** | **1** | **0** | **0** |
| Alagoas | 0 | 0 | 0 |
| Bahia | 0 | 0 | 0 |
| Ceará | 1 | 0 | 0 |
| Maranhão | 0 | 0 | 0 |
| Paraíba | 0 | 0 | 0 |
| Pernambuco | 0 | 0 | 0 |
| Piauí | 0 | 0 | 0 |
| Rio Grande do Norte | 0 | 0 | 0 |
| Sergipe | 0 | 0 | 0 |
| **Central-West** | **0** | **1** | **0** |
| Goiás | 0 | 1 | 0 |
| Mato Grosso | 0 | 0 | 0 |
| Mato Grosso do Sul | 0 | 0 | 0 |
| **Southeast** | **2** | **0** | **0** |
| Espírito Santo | 0 | 0 | 0 |
| Minas Gerais | 0 | 0 | 0 |
| Rio de Janeiro | 0 | 0 | 0 |
| São Paulo | 2 | 0 | 0 |
| **South** | **4** | **6** | **5** |
| Paraná | 3 | 0 | 0 |
| Rio Grande do Sul | 1 | 5 | 5 |
| Santa Catarina | 0 | 1 | 0 |

**
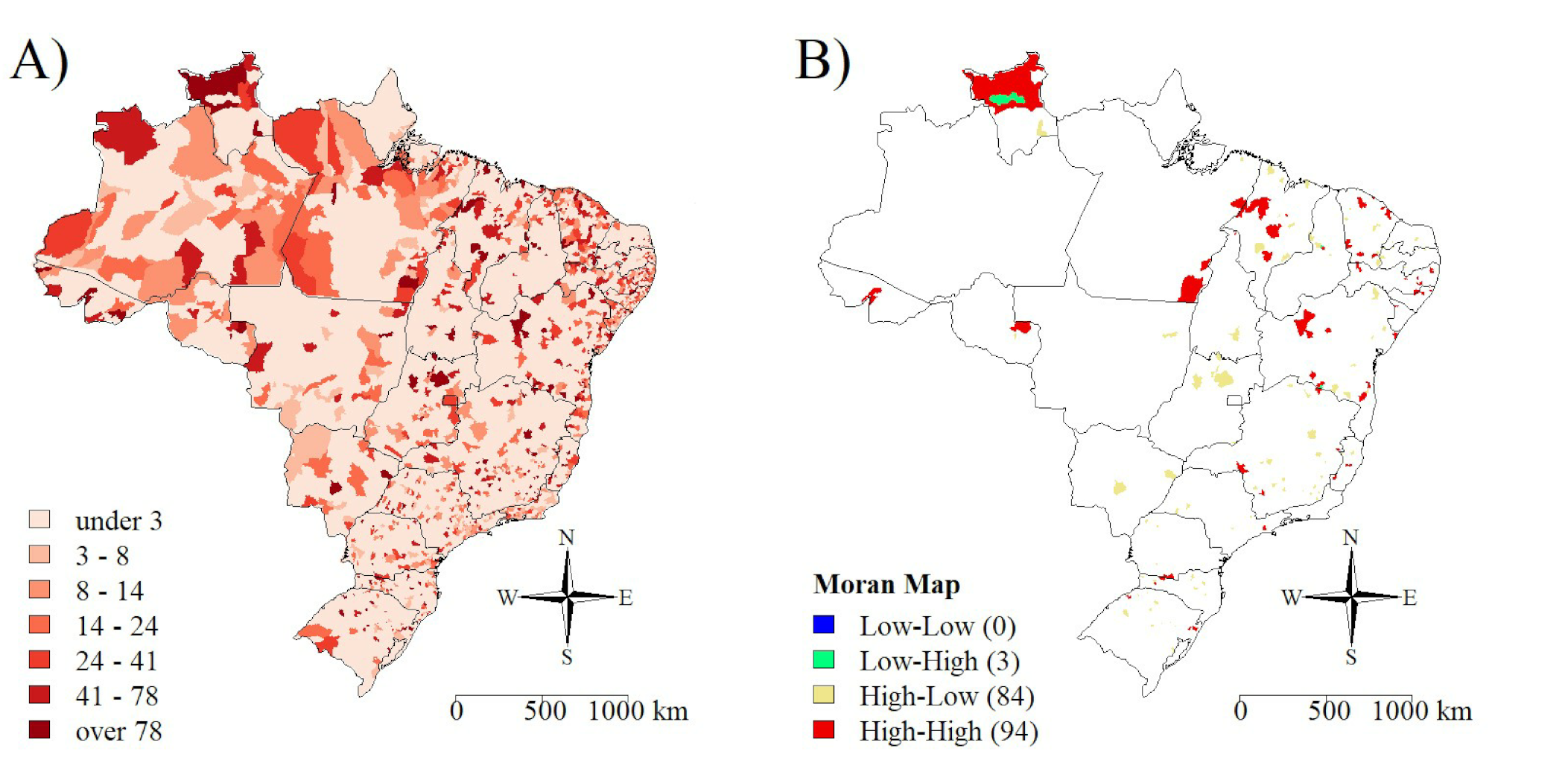
**

**Supplementary Figure 3. Spatial distribution of A) the COVID 19 case fatality rate per 100,000 children and adolescents aged 0-19 years and B) Moran Map for Children aged 0-19 years, in Brazil from March 2020 to October 2021.**

**Supplementary Table 5. Number of municipalities by Region/State by age group according to COVID-19 case fatality rate stratum.**

| **Age group/Region/State** | **Stratum** | | |
| --- | --- | --- | --- |
|  | **High-High** | **High-Low** | **Low-High** |
| **0-4 years old** | **No. municipalities** | **No. municipalities** | **No. municipalities** |
| **Brazil** | **64** | **84** | **2** |
| **North** | **17** | **6** | **2** |
| Acre | 0 | 2 | 0 |
| Amapá | 0 | 0 | 0 |
| Amazonas | 1 | 0 | 0 |
| Pará | 9 | 0 | 1 |
| Rondônia | 2 | 1 | 0 |
| Roraima | 5 | 1 | 1 |
| Tocantins | 0 | 2 | 0 |
| **Northeast** | **40** | **40** | **0** |
| Alagoas | 2 | 0 | 0 |
| Bahia | 7 | 10 | 0 |
| Ceará | 19 | 6 | 0 |
| Maranhão | 1 | 7 | 0 |
| Paraíba | 2 | 6 | 0 |
| Pernambuco | 9 | 2 | 0 |
| Piauí | 0 | 4 | 0 |
| Rio Grande do Norte | 0 | 3 | 0 |
| Sergipe | 0 | 2 | 0 |
| **Central-West** | **1** | **7** | **0** |
| Goiás | 1 | 5 | 0 |
| Mato Grosso | 0 | 2 | 0 |
| Mato Grosso do Sul | 0 | 0 | 0 |
| **Southeast** | **4** | **23** | **0** |
| Espírito Santo | 0 | 4 | 0 |
| Minas Gerais | 2 | 14 | 0 |
| Rio de Janeiro | 0 | 1 | 0 |
| São Paulo | 2 | 4 | 0 |
| **South** | **2** | **8** | **0** |
| Paraná | 0 | 0 | 0 |
| Rio Grande do Sul | 0 | 2 | 0 |
| Santa Catarina | 2 | 6 | 0 |
| **5-9 years old** |  |  |  |
| **Brazil** | **6** | **0** | **0** |
| **North** | **0** | **0** | **0** |
| Acre | 0 | 0 | 0 |
| Amapá | 0 | 0 | 0 |
| Amazonas | 0 | 0 | 0 |
| Pará | 0 | 0 | 0 |
| Rondônia | 0 | 0 | 0 |
| Roraima | 0 | 0 | 0 |
| Tocantins | 0 | 0 | 0 |
| **Northeast** | **6** | **0** | **0** |
| Alagoas | 0 | 0 | 0 |
| Bahia | 0 | 0 | 0 |
| Ceará | 1 | 0 | 0 |
| Maranhão | 0 | 0 | 0 |
| Paraíba | 0 | 0 | 0 |
| Pernambuco | 2 | 0 | 0 |
| Piauí | 0 | 0 | 0 |
| Rio Grande do Norte | 0 | 0 | 0 |
| Sergipe | 3 | 0 | 0 |
| **Central-West** | **0** | **0** | **0** |
| Goiás | 0 | 0 | 0 |
| Mato Grosso | 0 | 0 | 0 |
| Mato Grosso do Sul | 0 | 0 | 0 |
| **Southeast** | **0** | **0** | **0** |
| Espírito Santo | 0 | 0 | 0 |
| Minas Gerais | 0 | 0 | 0 |
| Rio de Janeiro | 0 | 0 | 0 |
| São Paulo | 0 | 0 | 0 |
| **South** | **0** | **0** | **0** |
| Paraná | 0 | 0 | 0 |
| Rio Grande do Sul | 0 | 0 | 0 |
| Santa Catarina | 0 | 0 | 0 |
| **10-14 years old** |  |  |  |
| **Brazil** | **11** | **0** | **0** |
| **North** | **5** | **0** | **0** |
| Acre | 0 | 0 | 0 |
| Amapá | 0 | 0 | 0 |
| Amazonas | 2 | 0 | 0 |
| Pará | 3 | 0 | 0 |
| Rondônia | 0 | 0 | 0 |
| Roraima | 0 | 0 | 0 |
| Tocantins | 0 | 0 | 0 |
| **Northeast** | **1** | **0** | **0** |
| Alagoas | 0 | 0 | 0 |
| Bahia | 1 | 0 | 0 |
| Ceará | 0 | 0 | 0 |
| Maranhão | 0 | 0 | 0 |
| Paraíba | 0 | 0 | 0 |
| Pernambuco | 0 | 0 | 0 |
| Piauí | 0 | 0 | 0 |
| Rio Grande do Norte | 0 | 0 | 0 |
| Sergipe | 0 | 0 | 0 |
| **Central-West** | **1** | **0** | **0** |
| Goiás | 1 | 0 | 0 |
| Mato Grosso | 0 | 0 | 0 |
| Mato Grosso do Sul | 0 | 0 | 0 |
| **Southeast** | **2** | **0** | **0** |
| Espírito Santo | 0 | 0 | 0 |
| Minas Gerais | 0 | 0 | 0 |
| Rio de Janeiro | 0 | 0 | 0 |
| São Paulo | 2 | 0 | 0 |
| **South** | **2** | **0** | **0** |
| Paraná | 0 | 0 | 0 |
| Rio Grande do Sul | 0 | 0 | 0 |
| Santa Catarina | 2 | 0 | 0 |
| **15-19 years old** |  |  |  |
| **Brazil** | **50** | **1** | **0** |
| **North** | **10** | **0** | **0** |
| Acre | 0 | 0 | 0 |
| Amapá | 0 | 0 | 0 |
| Amazonas | 2 | 0 | 0 |
| Pará | 7 | 0 | 0 |
| Rondônia | 0 | 0 | 0 |
| Roraima | 0 | 0 | 0 |
| Tocantins | 1 | 0 | 0 |
| **Northeast** | **23** | **1** | **0** |
| Alagoas | 4 | 0 | 0 |
| Bahia | 6 | 1 | 0 |
| Ceará | 5 | 0 | 0 |
| Maranhão | 4 | 0 | 0 |
| Paraíba | 0 | 0 | 0 |
| Pernambuco | 3 | 0 | 0 |
| Piauí | 0 | 0 | 0 |
| Rio Grande do Norte | 0 | 0 | 0 |
| Sergipe | 1 | 0 | 0 |
| **Central-West** | **0** | **0** | **0** |
| Goiás | 0 | 0 | 0 |
| Mato Grosso | 0 | 0 | 0 |
| Mato Grosso do Sul | 0 | 0 | 0 |
| **Southeast** | **8** | **0** | **0** |
| Espírito Santo | 0 | 0 | 0 |
| Minas Gerais | 0 | 0 | 0 |
| Rio de Janeiro | 3 | 0 | 0 |
| São Paulo | 5 | 0 | 0 |
| **South** | **9** | **0** | **0** |
| Paraná | 3 | 0 | 0 |
| Rio Grande do Sul | 4 | 0 | 0 |
| Santa Catarina | 2 | 0 | 0 |

**Supplementary Table 6. Spatial clusters of COVID-19 cases per 100,000 paediatric population (0-19 years) in Brazil from March 2020 to October 2021.**

| **Cluster #** | **State** | **No. Municipalities** | **Risk Relative** |
| --- | --- | --- | --- |
| 1 | Mato Grosso | 1 | 87·8 |
| 2 | Amapá | 1 | 30·0 |
| 3 | Santa Catarina | 1 | 11·1 |
| 4 | Mato Grosso | 5 | 9·0 |
| 5 | Mato Grosso | 3 | 8·5 |
| 6 | Mato Grosso | 1 | 8·5 |
| 7 | Amazonas | 6 | 8·2 |
| 8 | Ceará | 1 | 7·3 |
| 9 | Mato Grosso | 4 | 6·6 |
| 10 | Santa Catarina | 3 | 6·4 |
| 11 | Mato Grosso | 4 | 6·1 |
| 12 | Amazonas/Pará | 6 | 5·0 |
| 13 | Sergipe | 7 | 4·7 |
| 14 | Amazonas | 5 | 3·9 |
| 15 | Amazonas | 1 | 3·6 |
| 16 | Rio Grande do Sul | 7 | 3·5 |
| 17 | Pará/Mato Grosso | 4 | 3·3 |
| 18 | São Paulo | 1 | 3·2 |
| 19 | Tocantins | 8 | 3·2 |
| 20 | Minas Gerais | 6 | 3·1 |
| 21 | Sergipe | 8 | 3·0 |
| 22 | Rio Grande do Norte/Paraíba | 5 | 3·0 |
| 23 | Sergipe | 6 | 2·9 |
| 24 | Bahia | 1 | 2·8 |
| 25 | Ceará | 5 | 2·7 |
| 26 | Sergipe | 9 | 2·6 |
| 27 | Sergipe | 9 | 2·5 |
| 28 | Amazonas | 8 | 2·4 |
| 29 | Mato Grosso | 7 | 2·4 |
| 30 | Pará | 5 | 2·4 |
| 31 | Paraná | 5 | 2·4 |
| 32 | Alagoas | 4 | 2·3 |
| 33 | Paraná | 5 | 2·3 |
| 34 | Amazonas | 5 | 2·3 |
| 35 | Pará | 4 | 2·2 |
| 36 | São Paulo | 4 | 2·2 |
| 37 | Mato Grosso | 6 | 2·2 |
| 38 | Minas Gerais | 5 | 2·1 |
| 39 | Rio de Janeiro | 3 | 2·1 |
| 40 | Pará | 5 | 2·0 |
| 41 | Rondônia | 2 | 2·0 |
| 42 | Pará | 3 | 2·0 |
| 43 | São Paulo | 1 | 1·9 |
| 44 | Rio de Janeiro | 5 | 1·9 |
| 45 | Pará | 6 | 1·9 |
| 46 | Pernambuco | 1 | 1·8 |
| 47 | Pará/Amapá | 4 | 1·8 |
| 48 | São Paulo | 4 | 1·7 |
| 49 | Maranhão/Piauí | 2 | 1·6 |
| 50 | São Paulo | 1 | 1·6 |
| 51 | Rio de Janeiro | 1 | 1·5 |
| 52 | Bahia | 3 | 1·5 |
| 53 | Rio de Janeiro | 6 | 1·5 |
| 54 | Paraíba | 5 | 1·5 |
| 55 | São Paulo | 7 | 1·4 |
| 56 | Ceará | 1 | 1·4 |

**Supplementary Table 7. Spatial clusters of COVID-19 deaths per 100,000 paediatric population (0-19 years) in Brazil from March 2020 to October 2021.**

| **Cluster #** | **State** | **No. Municipalities** | **Risk Relative** |
| --- | --- | --- | --- |
| 1 | Sergipe | 9 | 3·9 |
| 2 | Bahia | 7 | 3·9 |
| 3 | Roraima | 8 | 3·5 |
| 4 | Rondônia/Amazonas | 3 | 3·3 |
| 5 | Ceará | 7 | 2·2 |
| 6 | Amazonas | 7 | 2·2 |
| 7 | Pernambuco | 7 | 2·2 |
