# Supplementary material for: Spatial clusters, social determinants of health and risk of COVID-19 mortality in Brazilian children and adolescents: A nationwide population-based ecological study
Source: Lancet Reg Health Am. 2022 Jun 29;13:100311. doi: 10.1016/j.lana.2022.100311 (PMC9242540; doi:10.1016/j.lana.2022.100311)
Supplement: Supplementary file 2 [file mmc2.docx]

**Editor note:** This translation in Portuguese was submitted by the authors and we reproduce it as supplied. It has not been peer reviewed. Our editorial processes have only been applied to the original abstract in English, which should serve as reference for this manuscript.

**Resumo**

**Antecedentes**

Dados sobre a distribuição geográfica dos casos e o risco de morte por COVID-19 em crianças e adolescentes são escassos. Este estudo descreve a distribuição espacial dos casos e mortes da COVID-19 na população pediátrica e sua relação com os determinantes sociais da saúde no Brasil.

**Métodos**

Trata-se de um estudo ecológico de base populacional com uma análise espacial de todos os casos e mortes devido à COVID-19 no Brasil entre crianças e adolescentes de 0-19 anos de março de 2020 a outubro de 2021. As unidades de análise foram os 5.570 municípios. Os dados sobre casos e mortes devido à COVID-19, vulnerabilidade social, desigualdades na saúde e capacidade do sistema de saúde foram obtidos a partir de bancos de dados disponíveis publicamente. Os municípios foram estratificados de baixa a muito alta incidência e mortalidade da COVID-19 usando procedimentos de agrupamento de meios K e agrupamentos espaciais, e os riscos relativos foram estimados usando estatísticas espaciais com modelos de probabilidade de Poisson. A relação entre as estimativas da COVID-19 e os determinantes sociais da saúde foi explorada usando técnicas de regressão Beta multivariada.

**Descobertas**

Um total de 33.991 casos de COVID-19 e 2.424 mortes entre crianças e adolescentes de 0 a 19 anos de idade foram registrados de março de 2020 a outubro de 2021. Houve uma dependência espacial para o coeficiente de mortalidade bruta por 100.000 habitantes na população pediátrica de 0-19 anos (I Moran 0-10; P < 0-001). Quarenta municípios tinham taxas de mortalidade mais elevadas, das quais 20 estavam em estados da região nordeste. Sete clusters espaciais foram identificados para a mortalidade da COVID-19, com quatro clusters na região Nordeste e três na região Norte. Municípios com maior desigualdade e vulnerabilidade social tiveram maior mortalidade da COVID-19 na população pediátrica.

**Interpretação**

Os principais grupos de risco de mortalidade entre crianças e adolescentes foram identificados nos municípios das regiões Norte e Nordeste, que são as regiões com os piores indicadores socioeconômicos e as maiores disparidades de saúde no país. Nossos resultados confirmaram a maior carga da COVID-19 para a população pediátrica brasileira em municípios maior desigualdade e vulnerabilidade social e piores indicadores socioeconômicos. Para reduzir a carga da COVID-19 sobre as crianças, é necessária uma imunização em massa.

**Financiamento:** Nenhum.
